# Factors influencing general practitioners’ decisions in migrant patients with mental health disorder

**DOI:** 10.1093/eurpub/ckac129.155

**Published:** 2022-10-25

**Authors:** C Duveau, C Wets, K Delaruelle, S Demoulin, M Dauvrin, B Lepièce, M Ceuterick, S De Maesschalck, P Bracke, V Lorant

**Affiliations:** 1 Institute of Research Health and Society, Université Catholique de Louvain, Woluwe Saint Lambert, Belgium; 2 Department of Sociology, Ghent University, Ghent, Belgium; 3 Psychological Sciences Research Institute, Université Catholique de Louvain, Louvain-la-Neuve, Belgium; 4 Belgian Health Care Knowledge Centre, KCE, Brussels, Belgium; 5 Department of Public Health and Primary Care, Ghent University, Ghent, Belgium

## Abstract

**Background:**

Patients with a migration background (MB) have more mental health disorders than those without migration background. Yet, those patients are still underrepresented in mental healthcare services and have more unmet medical needs. Although providers’ bias has been well studied, up to date, little is still known about the factors explaining those biases. We assessed the effect of general practitioners’ (GPs') individual and organizational factors on their decision-making regarding diagnosis, treatment and referral recommendations for patient with MB with symptoms of major depression.

**Methods:**

An experimental study staged a video-vignette of a depressed patient with or without MB. GPs had to make decision about diagnosis, treatment and referral. We then assessed the influence of several factors on their decisions such as age, ethnicity, workload and patient confidence. ANOVA and MANOVA were used for analyses.

**Results:**

Overall, we found more unfavourable decisions in GPs diagnosis and treatment recommendations regarding the patient with a MB (F = 3.56, p < 0.001). In addition, they considered the symptoms of the patient with a MB as less severe (F = 7.68, p < 0.01) and would prescribe less often a medical treatment to these patients (F = 4.09, p < 0.05). Nevertheless, few factors explained these differences, except the age, the workload and the patient trustworthiness.

**Conclusions:**

This paper highlighted GPs biases based on apparent migration background of a patient with major depression that perpetuates ethnic inequalities in mental health care. Further research into the origins of discrimination in primary mental health care are needed to explain how and when those discriminations arise.

**Key messages:**

• This paper shed light on pervasive unintentional discrimination still persist in mental health care among migrants in Europe.

• These findings may help us to further understand the role of general practitioner behaviour in primary mental health care discrimination.

